# The Impact of a Digital Digestive Health Program and Telehealth Visits in Socially Vulnerable Populations: Cohort Evaluation

**DOI:** 10.2196/70748

**Published:** 2025-09-11

**Authors:** Sanskriti Varma, Alicen Black, Erin Commons, Pen-Che Ho, Dena M Bravata, Hau Liu

**Affiliations:** 1 Division of Gastroenterology Massachusetts General Hospital Boston, MA United States; 2 Center for Neurointestinal Health Massachusetts General Hospital Boston, MA United States; 3 Harvard Medical School Boston, MA United States; 4 Cylinder Health Chicago, IL United States; 5 Stanford Medicine Stanford, CA United States; 6 Department of Medicine NYU Grossman School of Medicine New York, NY United States

**Keywords:** telehealth, health equity, gastrointestinal diseases, digestive symptoms, digital health

## Abstract

**Background:**

Socially vulnerable populations have less access to quality gastrointestinal (GI) care. Digital telehealth services provided by GI-focused registered dietitian nutritionists (RDNs) and digestive health coaches (HCs) may improve digestive health outcomes by facilitating access to GI care and thereby reduce health care disparities among vulnerable populations.

**Objective:**

The objectives of this study were to (1) evaluate the impact of a digital digestive health program on reducing GI symptoms among socially vulnerable populations and (2) assess whether telehealth visits with digital app use provide additional benefits in symptom reduction compared to digital app use alone among socially vulnerable populations.

**Methods:**

A comprehensive digital digestive care program with optional telehealth visits with RDNs and HCs was provided to US employees of participating companies via their employee benefits. We enrolled participants in the program between 2022 and 2023 who tracked digestive symptoms multiple times at baseline and then over 90 days. We measured changes in GI symptoms from baseline to up to 3 months, comparing those who opted for telehealth visits with those who used the app only. We stratified participants by the median Social Vulnerability Index (SVI) to evaluate symptom improvement across socially vulnerable populations. Multivariable regressions adjusted for age, gender, race, BMI, and preexisting GI conditions.

**Results:**

A total of 1656 participants met the inclusion criteria, of which 1362 (82%) scheduled at least one telehealth visit and 294 (18%) used only app-based resources. The majority (n=1417 86%) of participants saw GI symptom improvement, with an average reduction of 60% in symptom burden (*P*<.001). Participants who used telehealth services and the app had a reduction in symptoms 16% greater than that of app-only users (*P*=.01). High-SVI participants (ie, those with an SVI score above the median of 0.4, indicating greater social vulnerability) had a 22% greater reduction in GI symptom severity score than app-only high-SVI participants (*P*=.04).

**Conclusions:**

Digital health solutions may be an important resource in advancing equitable access to quality GI care and addressing disparities among populations with high social vulnerability. Virtual telehealth visits with RDNs and HCs appear to be particularly beneficial in improving digestive symptoms in such populations.

## Introduction

Chronic gastrointestinal (GI) diseases, including *Helicobacter pylori* infection, irritable bowel syndrome, pancreatitis, and celiac disease, are highly prevalent in the United States, accounting for high rates of mortality and cost that lead to significant burdens on health care systems, employers, and individual patients [1-3].

Despite this, less than 20% of individuals with abdominal pain, bloating, or diarrhea consult a health care provider for evaluation and management of these symptoms [4]. In this regard, telemedicine may offer increased access to GI specialty care and may also allow for standardized digital digestive care programs to provide effective long-term management options [5].

In addition to the high prevalence and economic burden of chronic GI diseases, considerable disparities exist in access to care across various populations. Patients with digestive diseases may experience gender, race/ethnicity, and economic disparities that lead to differential outcomes, including lower access to screening, lower education and health literacy, and poor food and nutrition [6-8]. For example, Black/African American patients face inequities in colon cancer screening rates, thought to be responsible for up to 42% of the disparity in colorectal cancer incidence and 19% of the disparity in mortality [9,10]. Historically vulnerable populations, including those with lower income, have low rates of telehealth adoption [11]. Structural factors contribute to disparities in telehealth access, including limited broadband access and lower rates of digital literacy [12].

We evaluated the impact of a digital app–based multimodal digestive health program (ie, virtual telehealth visits, app-based content, and tracking) on reducing GI symptoms in socially vulnerable populations. Telehealth visits with registered dietician nutritionists (RDNs) and health coaches (HCs) facilitate the building of trust and rapport between patients and their care team, develop new skills to manage GI issues, and encourage continuity of care. Thus, we sought to evaluate the improvement in chronic GI symptoms among participants who used telehealth visits with care from RDNs and HCs and app-based content compared to those who used app-based content alone.

## Methods

### Participants

The intervention has been described elsewhere [5,13]. Briefly, adult participants across the United States were provided access to Cylinder Health, a digital digestive chronic care program, through their employee benefits. Employer-approved marketing materials were used for outreach (eg, mailings and emails). Inclusion criteria included (1) preexisting GI diagnoses or symptoms; (2) enrollment in the program between January 1, 2022, and November 28, 2023; (3) symptoms recorded at baseline and on the app between days 30 and 90 after registration; and (4) symptom data recorded more than once. Access to the program was free for participants, who were not otherwise compensated for their participation.

### Data Collection

Survey administration and symptom tracking were performed within the app. Participants completed an intake survey at baseline, including information on demographics and GI history (eg, previously diagnosed GI conditions and digestive symptoms over the last week). Each participant was assigned a score on the US Centers for Disease Control and Prevention’s Social Vulnerability Index (SVI) based on their 5-digit zip code [14]. The SVI is presented as a percentile from 0 (least vulnerable) to 1 (most vulnerable) that allows geographies to be directly compared based on 16 measures across 4 categories per geography: socioeconomic status (rate of population below 150% of the federal poverty line, unemployed, high housing cost burden, no high school diploma, no health insurance), household characteristics (percentage aged 65 years or older, aged 17 years or younger, civilian with a disability, single-parent households, and English language proficiency), racial and ethnic minority status, and housing type and transportation (rate of multiunit structures, mobile homes, crowding, no vehicle, and group quarters). We collected information on racial group from the patients directly, so we did not use racial information from the SVI. To compare participants on the basis of their social vulnerability, we categorized participants as having high social vulnerability if their SVI score was greater than the median (0.4) and low social vulnerability if their SVI was less than or equal to the median. We then report data for groups stratified into those above or below the median SVI.

GI symptom tracking in the program has been described elsewhere [5,13]. Participants provided information on their digestive symptoms over the prior week and tracked symptoms via the app. Participants were asked to rate 9 common GI symptoms (abdominal pain, bloating, diarrhea, constipation, reflux, gas, nausea, vomiting, and loss of bowel control) on a 5-point scale: 0 (no symptoms), 1 (mild symptoms), 2 (moderate symptoms), 3 (severe symptoms), and 4 (very severe symptoms). Scores for each individual symptom and a composite symptom score (0 to 36, computed as the sum of each of the scores) were determined at baseline (prior to program engagement) and at the time of the last recorded symptom between days 30 and 90 of program participation.

### Intervention

All participants used app-based content and were provided access to telehealth, coaching, and nutrition services. Participants who used the telehealth and app services (“telehealth+app”) were compared to participants who used the app alone (“app-only,” defined as all aspects of the program except the synchronous telehealth visits). At the time of this study, the app was available only in English; however, a real-time translation service was available to support telehealth visits in more than 200 languages. If participants used the app 3 or more times to track symptoms, they were considered frequent symptom trackers.

RDNs provided individualized, evidence-based, and culturally sensitive nutrition guidance [15-17]. Similarly, GI HCs provided individualized support with goal setting, lifestyle management (eg, stress reduction, mindfulness, healthy sleep, and physical activity), medication adherence, self-advocacy, self-monitoring, coach-led cognitive behavioral therapy, and assistance with the app. The program delivered via the app provides each participant with a personalized care plan that includes targeted education on their conditions, symptoms, and supportive lifestyle interventions. If participants had any telehealth interaction with either an RDN or HC, we classified them as having used telehealth.

### Statistical Analysis

We compared means and proportions at baseline for each of the participant characteristics (age, gender, race, BMI, and SVI) using the Student *t* test and chi-square test. We computed the change in the average GI symptom severity score from the baseline score to the latest recorded score for the 2 groups: telehealth+app and app only. The GI symptom severity score ranges from 0 to 36 (maximum 4 points per symptom for 9 GI symptoms).

To evaluate for clinically significant symptom changes during the program, we performed a logistic regression analysis to evaluate the probability that participants at presentation had multiple (2 or more) severe or very severe symptoms and then reported such symptoms as mild or none at program end, controlling for age, gender, race, baseline BMI, days between registration and last check, and baseline total symptom score. Although we did not control for program engagement (eg, symptom tracking), we did control for days between registration and last check, which is a proxy for tracking frequency.

To control for potential confounding factors, the average treatment effect for the treated (ATT) of the severity score change was computed through regression adjustment with the following covariates: age, gender, race, baseline BMI, baseline GI symptom severity scores, and number of days between program registration and the latest recorded score. We performed this analysis using the CAUSALTRT approach in SAS (SAS Enterprise Guide; version 8.4), which estimates the ATT based on the predicted symptom severity score change from generalized linear models fitted for the telehealth+app and the app-only groups separately. *P* values <.05 were considered statistically significant.

### Ethical Considerations

Given that all data were deidentified and routinely collected as part of the condition management program, this protocol, based on secondary analyses of existing data, was considered exempt by the WCG institutional review board (VORD.00A; April 2023). Participants were provided access to the digestive health program via their employer benefits but were not otherwise compensated for participation.

## Results

### Participant Characteristics

Overall, 1656 participants completed the registration process and were enrolled in the study (Table 1). The average age of the participants was 43 (SD 12) years; 75% (n=1239) identified as female, 67% (n=1100) identified as White/Caucasian, 11% (n=187) as Asian/Pacific Islander, 6% (n=105) as Latino/Latina, 7% (n=120) as Black, and 9% (n=144) as being multiple/other races (Table 1). On average, participants were overweight (mean BMI 29, SD 7). The median SVI was 0.4 (SD 0.2). When stratified by median SVI (<0.4 vs >0.4), the high-SVI group (mean SVI 0.6, SD 0.1) was more likely to be female (n=632, 79% vs n=607, 71%) and less likely to be White/Caucasian (n=518, 64% vs n=582, 68%) than the low-SVI group (mean SVI 0.3, SD 0.1).

**Table 1:** Participant characteristics, stratified by Social Vulnerability Index (SVI)^a^.

Characteristic		Total (N=1656)	High SVI (>0.4; n=805, 49%)	Low SVI (<0.4; n=851, 51%)	*P* value	
Age (years), mean (SD)		43 (12)	43 (12)	43 (11)	.50	
**Gender, n (%)**						<.001
	Female	1239 (75)	632 (79)	607 (71)		
	Male	408 (25)	166 (21)	242 (28)		
	Prefer not to disclose	9 (1)	7 (1)	2 (0.2)		
**Race, n (%)**						<.001
	African-American/Black	120 (7)	87 (11)	33 (4)		
	Asian/Pacific Islander	187 (11)	61 (8)	126 (15)		
	Latino/Latina	105 (6)	67 (8)	38 (5)		
	Multiple/other	144 (9)	72 (9)	72 (8)		
	White/Caucasian	1100 (67)	518 (64)	582 (68)		
SVI, mean (SD)		0.4 (0.2)	0.6 (0.1)	0.3 (0.1)	<.001	
**BMI (kg/m^2^)**						<.001
	Overall, mean (SD)	29 (7)	29 (7)	28 (7)		
	<18.5, n (%)	25 (2)	17 (2)	8 (1)		
	≥18.5 to <25, n (%)	539 (33)	224 (28)	315 (37)		
	≥25 to <30, n (%)	529 (32)	253 (31)	276 (32)		
	≥30, n (%), n (%)	563 (34)	311 (39)	252 (30)		
Change in total symptom score, mean (SD)		–4.5 (4)	–4.7 (5)	–4.4 (4)	.10	

^a^The SVI ranges from 0 to 1, with higher scores indicating higher risk for social vulnerability. The median score was 0.4 for this population.

### Telehealth Use

The majority of participants (n=1362, 82%) had at least one telehealth visit with a member of their care team and were in the telehealth+app group. Just 18% (n=294) of participants used the app only. Among those participants in the telehealth+app group, 63% (n=859) were frequent symptom trackers. In contrast, among those participants in the app-only group, 43% (n=127) were frequent symptom trackers. There was no difference between the high- and low-SVI groups in the rate of frequent symptom trackers (n=478, 59.4% vs n=508, 59.7%; *P*=.90).

### Symptom Improvement

The majority (n=1417, 86%) of participants reported improvement in their total GI symptom score, with an average reduction of 60% in their total symptom score (*P*<.001) relative to baseline. At baseline, the most commonly reported symptoms were gas (n=1545, 93%), bloating (n=1387, 84%), constipation (n=888, 54%), and abdominal pain (n=880, 53%) [5]. On average, participants reported a statistically significant improvement for all symptoms (*P*<.05 for each symptom) [5]. At baseline, 80% (n=1316) of participants reported moderate or severe GI symptom severity for at least one symptom. In contrast, at the end of the intervention, only 47% (n=784) of participants reported moderate or severe symptoms and 16% (n=270) reported no symptoms.

Telehealth+app participants’ symptom reduction was 16% (0.7) greater than app-only participants (*P*=.01). High-SVI participants (median score >0.4) showed a 22% greater reduction in total GI symptom score among telehealth+app participants compared to app-only participants (*P*=.04). The clinical impact of telehealth visits in those with high social vulnerability was particularly notable in participants presenting with higher symptomatic acuity. High-SVI participants who were in the telehealth+app group and reported multiple severe or very severe digestive symptoms at baseline were significantly more likely to report no symptoms or only mild symptoms at the end of the program compared to the app-only group (odds ratio [OR] 3.6, 95% CI 1.15-11.26). In contrast, low-SVI participants who were in the telehealth+app group and reported multiple severe or very severe symptoms at baseline were not more likely than those in the app-only group to report symptom resolution or just mild symptoms (OR 0.3, 95% CI 0.09-1.12).

Overall, telehealth+app participants had significantly reduced GI symptom severity scores relative to app-only participants (mean change –0.65, 95% CI –0.13 to –1.17; *P*=.01). The ATT of the high-SVI group was over twice the ATT of the low SVI group (mean change –0.87, 95% CI –0.04 to –1.69; *P*=.04 vs mean change –0.39, 95% CI 0.18 to –0.96; *P*=.18).

## Discussion

### Principal Findings

This study had two key findings. First, participation in the digital digestive care management program was associated with a reduction in GI symptoms for all groups, including the entire population and those with both low and high SVI. We have previously reported similar findings showing that participants of all genders, races, and SVI strata experienced significant, comparable improvement in GI symptoms [13]. A digital GI care management program, including telehealth visits with RDN and HC support, can be an effective approach for improving debilitating GI symptoms. The strengths of this analysis include, first, the relatively large sample size of nearly 1700 participants followed longitudinally; the richness of the demographic data collected on participants (novel for digital health interventions), including race/ethnicity information and geographic location, which enabled detailed analyses of historically vulnerable populations; and use of the validated SVI to characterize social vulnerability among people with chronic digestive issues.

Second, the additional use of telehealth visits with RDNs and HCs significantly increased symptom reduction for all participants, especially so for participants with higher social vulnerability. This suggests that a multimodal digestive care management program, specifically one incorporating longitudinal synchronous human interaction in addition to digital tools, may be relevant to users with GI symptoms and could have an impact on improving health equity, particularly in those with high social vulnerability. Patients with higher social vulnerability often have poorer access to care, lower health literacy, and worse clinical outcomes; therefore, improving outcomes in this group is an active area of research and policy implementation [18]. Physical access to care is thought to be the most substantial obstacle to health equity, related to low physician supply in rural areas and lack of vehicle access in households of low socioeconomic status. Telehealth visits may bridge the gap by increasing access to culturally competent health care practitioners, as such access is crucial to meeting each individual’s unique needs and improving outcomes with the increase in racial and ethnic diversity in the United States [19]. Finally, telehealth synchronous visits allow for regular follow-up and continuity of care, which is crucial for effective treatment, preventive care, psychosocial care, and chronic disease management, potentially reducing health care fragmentation [20,21]. It is plausible that the high-SVI participants in our cohort may have experienced these benefits of telehealth, ultimately contributing to the significant improvement shown with our telehealth program. Given the rise of artificial intelligence–based assistants for virtual care, future studies should evaluate whether the human touch experienced by participants in this study could be as effectively delivered by such novel technology. Notably, more participants in the telehealth+app group were frequent symptom trackers than those in the app-only group (63% vs 43%). Symptom tracking may be a marker for greater engagement in self-care, which could have contributed to the improved outcomes in the telehealth+app group. Prior studies in other clinical disciplines have demonstrated that symptom tracking is strongly associated with higher rates of adequate self-management practices that can improve quality of life [22], prevent clinical deterioration [23], and reduce preventable hospitalizations [24]. However, since there was no difference in the rate of frequent symptom trackers between the high- and low-SVI groups, the observed differences within the telehealth+app group by SVI are unlikely to be associated with symptom tracking frequency.

### Future Work

These promising findings warrant additional study to further explore the role of digestive telehealth for vulnerable populations. Since participants self-selected to use telehealth plus the app versus using the app alone, it may have been that participants who used the app only had poorer internet access, digital literacy, patient activation, or other key predictors of health outcomes. Internet access may be a particularly important confounder that was not specifically evaluated in this study (although telehealth visits were available both via video and over the phone). Also, the participants with higher social vulnerability in this study had commercial insurance, and all were at least able to use the app or web browser version of the service; thus, they may have had more advantages than other populations with similar SVI scores. A future evaluation of this digital digestive care program for socially vulnerable populations with other forms of insurance (eg, Medicaid) could expand our understanding of the utility of a digital digestive care management program for low-income groups.

### Limitations

Our study has four key limitations. First, given the design of this study, we did not have the ability to control for unmeasured confounding factors such as health motivation and literacy, symptom severity, access to broadband, and language barriers. Given that participants self-selected for the use of virtual care, it limits the ability to infer causality between engagement with the program and outcomes. Second, we did not assess types of additional care that participants may have been receiving, such as external primary care or specialty care. Third, the study population only included commercially insured adults, which may limit the generalizability of these results to other populations. However, our findings may be further amplified in populations at higher social vulnerability risk (eg, Medicaid or Medicare populations). Finally, this study was funded by Cylinder Health, which provided the service evaluated. Notably, the authors adhered to best practices for data analysis and interpretation and report results in accordance with the STROBE (Strengthening the Reporting of Observational studies in Epidemiology) guidelines (Multimedia Appendix 1).

### Conclusions

A multimodal digital digestive care program that includes synchronous telehealth visits with RDNs and HCs can improve digestive symptoms in a diverse commercially insured population and may be a promising tool for increasing access and improving clinical outcomes for populations with high social vulnerability who have chronic GI symptoms. Future research should assess the effects of virtual digestive care programs in providing long-term effective care to those affected by health care disparities.

## Appendix

Multimedia Appendix 1 STROBE checklist.

## Abbreviations

ATT: average treatment effect for the treated
GI: gastrointestinal
HC: health coach
OR: odds ratio
RDN: registered dietitian nutritionist
STROBE: Strengthening the Reporting of Observational studies in Epidemiology
SVI: Social Vulnerability Index

## Data Availability

The datasets generated or analyzed during this study are available from the corresponding author on reasonable request.
